# Reviewers acknowledgement

**DOI:** 10.1002/1878-0261.12892

**Published:** 2021-01-04

The Editors of *Molecular Oncology* would like to thank all referees who have given their time and expertise to review articles submitted for publication in Volume 14. *Molecular Oncology* heavily relies on reviewers' careful assessment, constructive criticism and timely response to ensure both quality and fast turnaround times.

The names of these reviewers are listed below; we apologize if we have inadvertently omitted anyone.

Athie Alejandro

Enriquez‐Navas Pedro M.

Fenical William

Ishimoto Takatsugu

Javaherian Kashi

Jensen Kenneth

Jungwirt Ute

LaBarge Mark

Montemurro Filippo

Mostoslavsky Raul

Nam Eun Ji

Utoft Niemann Carsten

Acosta Juan‐Carlos

Addeo Alfredo

Affar El Bachir

Agoulnik Alexander I.

Ahirwar Dinesh

Ahmad Shiekh Tanveer

Ahmed N.

Ahmed Zeeshan

Akbari‐Birgani Shiva

Akiyama Yoshimitsu

Alexandraki Krystallenia

Alfieri Roberta

Almeida Raquel

Almonte Maribel

Alqathama Aljawharah

Altucci Lucia

Alus Xiaoli

Ambros Peter F.

Amit M.

Andersen Rikke F.

Andl Claudia D

Anfossi Simone

Apetrei Cristian

Arabi Seyed Mostafa

Arcangeli Annarosa

Ariey‐Bonnet Jérémy

Arlt Alexander

Arteaga Maria‐Francisca

Asangani Irfan

Ashktorab Hassan

Aspenström Pontus

Audet‐Delage Yannick

Avery V.

Avril Stefanie

Aydin Omer

Azencott Chloé‐Agathe

Azizi Masoumeh

Baba Hideo

Baldassarre Gustavo

Balusu Ramesh

Baniahmad Aria

Bao Xuanwen

Barderas Rodrigo

Barr Frederic

Barrett John W.

Barsyte Dalia

Bartel Karin

Barton Michael

Bartsch Joerg Walter

Basu Moitri

Bauerschlag Dirk

Baxter Robert C.

Bayraktar Recep

Beaumatin Florian

Beemon Karen

Belkhiri Abbes

Belletti Barbara

Bellucci Roberto

Bernardo Carina

Bernards Rene

Bernassola Francesca

Berns Anton J.M.

Bertucci François

Biamonte Flavia

Birch Joanna L.

Birner‐Grünberger Ruth

Bishai William R.

Black E. Penni

Blandino Giovanni

Bleckmann Annalen

Blum Yuna

Boegel Sebastian

Boerma Marjan

Boncela Joanna

Bonci Desiree

Borella Fulvio

Borg Ake

Borkowska Edyta

Borrego Francisco

Bose Chinmoy

Bosisio Francesca

Bossis Guillaume

Bou‐Dargham Mayassa

Boulagnon‐Rombi Camille

Bourette Roland P.

Bovee J.V.

Boye Kjetil

Bravaccini Sara

Brody Jonathan

Brown Bob

Brustugun Odd Terje

Budanov Andrei

Budczies J.

Bullova Petra

Burdette Joanna

Burnstein Kerry L.

Büttner Reinhard Büttner

Cabeçadas José

Cai Changmeng

Cai Hao

Cai Gengming

Calin George A.

Cao Shun‐wang

Caraglia Michele

Cardona Andres F.

Carneiro Fatima

Carnero Amancio

Carpenter Richard

Carpenter Valerie J.

Carretta Chiara

Carroll Bernadette

Casal J. Ignacio

Castelo‐Branco P.

Castillo‐Lluva Sonia

Cava Claudia

Cejas Paloma

Ceraline Jocelyn

Cervantes Andres

Cetrulo Curtis L.

Cexus Olivier

Chada Kiran

Challagundla Kishore Babu

Chan Edmond

Chang Guoqiang

Chang Suhwan

Chang Wei‐Chao

Charan Manish

Chatterjee Samit

Chatzaki Ekaterini

chen BaoQing

Chen Eleanor

Chen Gang

Chen Hui

Chen Jung‐Tsu

Chen Leilei

Chen Li‐Han

Chen Rong

Chen Wei

Chen Yaohui

Chen Zhe‐Sheng

Chen Zhicong

Cheng De

Cheng Shiyuan

Chern Yi‐Jye

Chia KeeMing

Chikuma Shunsuke

Chiu H.A.

Cho Je‐Yoel

Cho Ssang‐goo

Chou Chih‐Hung

Christina Voelkel‐Johnson

Church David

Citron Francesca

Clarke Robert

Classens F.

Coffelt Seth

Cohen Adam

Collis Spencer

Cong Yu‐Sheng

Cooper Wendy

Corey Eva

Corrado Giacomo

Corso Simona

Cortot Alexis

Costa Jose Luis

Costanzo Vincenzo

Costello Joseph

Cottu Paul H.

Coulouarn Cedric

Crea Francesco

Cuezva Jose M.

Culig Zoran

Daidone Maria Grazia

Dalvi Priya

Das Dipon

Davalos Albert

Davidson Ben

De Bortoli Michele

de Langen A.J.

De Lorenzo Francesco

De los Santos Mireia Cruz

Dedhar Shoukat

Delannoy Philippe

Dellis Olivier

Deming D.A.

Demonacos Constantinos

Demoulin Jean‐Baptiste

Désert Romain

Deubzer Hedwig E.

Diamandis Eleftherios

Didazio Pietro

Diederichs Sven

Dika Emi

Dillon Richard

Dimberg Anna

Dimopoulos Konstantinos

Ding Bisen

Ding Ling‐Wen

Dive Caroline

Domany Eytan

Dong Jun

Dong Zhong‐Yi

Donia Marco

Dos Santos Camila O.

Dragomir Mihnea P.

Dreyer S.B.

Driescher Caroline

Dubbink Hendrikus J.

Dumbrava Ecaterina Ileana

Dunlop Elaine

Duran Raul V.

Dyer M.

Eder Iris E.

Eferl Robert

Ehata Shogo

Eiring Anna

Ekert Paul

Elgamal Sara

Engle Danielle

Enserink Jorrit

Erin N.

Espina Carolina

Fabbri Muller

Fabbris Linda

Faller William James

Fan Qingxia

Fang Bingliang

Fang Weiyi

Fernandez Sandra

Ferretti Elisabetta

Fijneman Remond

Filomeni Giuseppe

Fitzgerald R.C.

Flanagan James M.

Fleischer Thomas

fleischhacker michael

Flouriot Gilles

Foersch Sebastian

Fokas Emmanouil

Fon tacer Klementina

Font de Mora Jaime

Forman David

Fornari Francesca

Fouassier Laura

Frank Malene

Fraser Michael

Gaber Ola

Gabrielli Brian

Gagliano Teresa

Gaipl Udo S.

Galán‐Moya Eva

Gallipoli Paolo

Ganapathy Vadivel

Ganguly Anutosh

Garbati Patrizia

Garlanda Cecilia

Germano Giovanni

Ghantous Akram

Giaccone Giuseppe

Giacomini Patrizio

Gikas Evangelos

Gillies Robert J.

Giovannetti E.

Gires Olivier

Gjertsen B.T.

Gobel Andy

Godoy‐Ortiz Ana

Goff Stephen P.

Goldberg Michal

Gomes Catarina

Gonçalves Emanuel

Gonzalez‐Martin Alice

Gopalakrishnan Dharmesh

Gourni Eleni

Gozuacik Devrim

Graber Tyson

Grabsch H.I.

Gramantieri Laura

Gregory Philip

Greinert Rüdiger

Griffoen A.W.

Grimm Elizabeth

Gromova Irina

Gross Julia

Grossman H. Barton

Guenes Cagatay

Guldberg Per

Gullberg Donald

Gungor C.

Guo Chang‐An

Guo Jessie

Guo Lvhua

Gupta Sonal

Gutierrez Alejandro

Haffner Michael

Hahn H.

Haiqian Liang

Halbrook Chris

Halezonetis Thanos D.

Hamerlik Petra

Han Jiali

Han Susu

Hanash S.M.

Hansen Torben F.

Harrell Joshua

hawse John

Hayashi Yujiro

Hayes Daniel F.

Hayes Liza M.

He Yongfeng

Hedman HÃ¥kan

Heining Christoph

Heitzer Ellen

Helgason Vignir

Helland Aslaug

Hellström Mats

Henriksson Roger

Henrique R.

Herbert Katharine

Herceg Zdenko

Herlyn Meenhard

Hermansen Niels Emil Ulrich

Heßmann Elisabeth

Holding Andrew

Hong Chibo

Hoon Dave

Hopper John L.

Hsiao Michael

Hu Huimin

Hu Yi‐Lin

Huang Huocong

Huang Jian

Huang Wei‐Chien

Huang Zhaohui

HuangYang Meng

Huehnchen Petra

Hughes Thomas A.

Humpton Timothy

Hunter Jill

Hyo Sup Shim

Ichim Gabriel

Iorio Francesco

Izbicki Jakob

Jabbarzadeh Kaboli Parham

Jaffe Elaine S.

Jansen Maurice

Janz Siegfried

Jeffrey Stefanie S.

Jin Zhe

Joerger Andreas

Józsi Mihály Krisztián

Jücker Manfred

Juhlin Carl Christofer

Juszczynski Przemyslaw

Kaipparetu Benny

Kamal Maud

Kang Byoung Heon

Kantelhardt Eva Johanna

Kashani‐Sabet Mohammed

Kataoka Naoyuki

Katara Gajendra Kumar

Katayama Ryohei

Kaushik Vivek

Kazi Julhash U.

Ke Kun

Keeshan Karen

Kelm Jens

Kenneth Niall S.

Kim Hangun

Kim Se Hoon

Kim Yonghyun

Kittas Chistos

Klec Christiane

Klomp Dennis

Knutsen Erik

Knuutila Sakari

Koehler Bruno

Koinuma Daizo

Koizume Shiro

Kolluri Siva Kumar

Kong Qing‐Peng

Korkaya Hasan

Korreman Stine

Kosari Farhad

Koskinen Paivi J.

Kruyt Frank A.E.

Kryza Thomas

Kube Dieter

Kuhlmann Jan Dominik

Kulasinghe Arutha

Kusebauch Ulrike

kwon young‐guen

Lammert Frank

Landergren Ulf

Landero‐Huerta Daniel

Lazar Vladimir

Lazo Pedro A.

Le Joncour Vadim

Lee In Hye

Lee Jenny

Lenk Lennart

Leszczynska Katarzyna

Li Heming

Li Ming

Li Shuren

Li Weidong

Li Zhihua

Liang Yongye

Liang Zhixian

Lianidou Evi

Liao Tsai‐Tsen

Liao Wen‐ting

Liao Xudong

Lin Aifu

Lin Jung‐Chun

Lin Yi

Lindbjerg Andersen Claus

Liou Geou‐Yarh

Liu Yanqing

Liu Zeyi

Liu Zhonghua

Lofgren Kristopher

Lohberger Birgit

Lohr Matthias

Lok Benjamin

Lopez Gonzalo

Lu Weiqin

Lu Yuanyuan

Lucas Clair

Lueckerath Katharina

M Bispo de Jesus

Ma Yun‐Feng

Ma Stephanie

Ma Yibao

Madak‐Erdogan Zeynep

Madeddu Roberto

Mælandsmo Gunhild Mari

Maghnouj Abdel

Magnani Diogo

Mai Chun‐Wai

Malapelle Umberto

Mansure Jose Joao

Mantovani Giovanna

Mao Xinliang

Marchetti Dario

Marcus Damiënne

Marini Alberto

Maritza Jaramillo

Marone Gianni

Marrone Kristen

Mateo Joaquim

Matheu Ander

Mathews Michael B.

Matino David

Mavila Nirmala

McCart Reed Amy

McCaughan Frank

McEwan David

McEwan Iain

Meder Lydia

Medova Michaela

Mehues Filip

Melino Gerry

Mezencev Roman

Michea Paula

Mikkelsen Jakob Giehm

Miller Mark Steven

Mineharu Yohei

Mineo Marco

Minsky Bruce

Mitra Kasturi

Miyamoto Hiroshi

Miyazawa Keiji

Mizuno Shohei

Modesti Mauro

Moestue Siver A.

Mondaca Sebastian

Morgan Iain

Morgenstern Milana

Mori T.

Morris Van

Mostert Bianca

Moustakas Aristidis

Mueller Miryam

Muinelo‐Romay Laura

Murphy Daniel

Murtaza Muhammed

Muzumdar Mandar Deepak

Naidu Samisubbu R.

Nakamura Tomoe Y.

Nakshatri Harikrishna

Nasr Rihab

Nasser Mohd Wasim

Nawaz Muhammad

Neesse Albrecht

Nelkin Barry D.

Ngo Vu Nguyen

Ni Jie

Ní Chonghaile Tríona

Nicolai Sara

Nie Chunlai

Nishihara Hiroshi

Nishitani Hideo

Nogueira Clotilde Costa

Nystrøm Håkan

Ohba Motoi

Ohtsuka MAsahisa

Okamoto Tatsuro

Olsson Ann

Opasic Luka

Ordoñez Jose Luis

Oren Moshe

Orimo Akira

Osborne Timothy

Osorio Hugo

Pabla Navjot

Palacios Jose

Palmieri Dario

Palmieri Giuseppe

Pandiella Atanasio

Parolia Abhijit

Passaro Antonio

Peng Guangyong

Perakis Samantha

Peruzzi Barbara

Petrescu George

Petrilli Alejandra

Philpott Mike

Pierga Jean‐Yves

Pivarcsi Andor

Planck Maria

Po Agnese

Poggi Alessandro

Pojo Marta Sofia

Pradeu Thomas

Prasmickaite Lina

Prieske Katharina

Prudovsky Igor

Pucci Perla

Pulinilkunnil Thomas

Qi Lishuang

Qiao Jianling

Radosa Julia

Rai Kammei

Rai Taranjit

Rak Janusz

Ralph Stephen J.

Raman Venu

Ramaswamy Vijay

Rameshwar Pranela

Ramírez de Molina Ana

Rappoport Joshua

Raychaudhuri Pradip

Reeh Matthias

Reid Glen

Ren Ning

Renner Kathrin

Reyes‐Santias Francisco

Rhost Sara Johanna

Ricklefs Franz

Riethdorf Sabine

Riggio Marina

Ringborg Ulrik

Rishi Arun K.

Ritterhouse Lauren L.

Rizos Helen

Robbins Hilary

Roesch Alexander

Roig‐Carles David

Romancer Muriel Le

Rosell Rafael

Rosenfeld Anatoly B.

Rosenfeldt Mathias

Rosonina Emanuel

Rospo Giuseppe

Rudenko Viktoria

Ryan Kevin

Rytelewski Mateusz

Saitoh M.

Sakamaki Jun‐ichi

Sakamoto Takeharu

Salgado‐Albarran Marisol

Sánchez‐Margalet Victor

Sancisi Valentina

Sander Sandrine

Santoni‐Rugiu Eric

Sarkar Devanand

Sasano Hironobu

Sauter Guido

Savaraj Niramol

Sayan Emre

Schaider Helmut

Schambach Axel

Schlüter Hartmut

Schmitt Fernando C.

Schoedel Johannes

Schott D.S.

Schroth Werner

Schulte Gunnar

Schumacher Johannes

Schuringa Jan Jacob

Schuuring Ed

Schüz Joachim

Schweiger Michal R.

Seki Naohiko

Sekido Yoshitaka

Sengupta S.

Seno Masaharu

Seshagiri Somasekar

Seufferlein Thomas

Shalu Jhanwar

Sharan Shyam

Sharma Jaspreet

Sharma Aarushi

Sheltzer Jason

Shen Yuehong

Shepherd Trevor

Shi Qihui

Shih Andres

Shih Jing‐Wen

Shukla Sudhanshu

Shurin Michael R.

Sikora Ewa

Silwal‐Pandit Laxmi

Singh Shiv

Sirbu Ovidiu

Sjöström Martin

Skoda Jan

Smirnov Artem

Sodir Nicole

Solinas Cinzia

Soltani Madjid

Son Jaekyoung

Song Libing

Soto Ubaldo

Soucek Laura

Speel Ernst Jan

Spindler Volker

Sproll Christoph

Stanton Benjamin

Staples Christopher

Stathopoulos G.T.

Stebbing J.

Stecca Barbara

Steffens Maryke

Steindorf Karen

Stern J.

Stoecklein Nikolas H.

Stoecklein Nikolas Hendrik

strappazzon Flavie

Strelnikov Vladimir

Su Changqing

Subramanian Janakiraman

Suenaga Yusuke

Sugimura Haruhiko

Sun Cheng‐Cao

Sun Jun

Sun Shi‐Yong

Sun Xiao‐Feng

Supek Fran

Surralles Jordi

Sutherland Kathe

Taghizadeh Hossein

Tahara Hidetoshi

Tahiri Andliena

Tai Xiang

Tait Stephen

Takayama Kenichi

Tan Wanlong

Tanaka Yuichiro

Tang Damu

Tang Hailin

Tarin David

Taubert Helge

Taubert Stefan

Tee Andrew

Tekpli Xavier

Telleria Carlos M.

Tennant Daniel

Teppan Julia

Tessaro Francesca

Testa Joseph

Thorburn Andrew

Tie Jeanne

Togawa Kayo

Tomlinson Michael G.

Tora MS

Tost Jorg

Tran Phuoc T.

Troncone Giancarlo

Trouw Leendert A.

Tsao Hensin

Turcan S.

Tyagi Abhishek

Tyner Jeff

Tzingounis Anastasios

Ulivi Paola

Umansky Viktor

Untergasser Gerold

Urciuoli Enrica

Vadlamudi R.K.

van der Heijden Michiel

van der Leest paul

van der Schaaf Arjen

Vandamme Timon

Vanden Berghe Wim

Vandenbrouck Yves

vanlandewijck michael

Vaquero Javier

Vaubel Rachel

Vergnaud Juliette

Vermeulen Louis

Vibhakar Rajeev

Viñals Francesc

Visco Carlo

Viviani Marco

Vlahou Antonia

Vo Dat

Voena Claudia

Vogelzang Nicholas

von Amsberg Gunhild

Vousden Karen H.

Vymetalkova Veronika

Wan Guohui

Wang Shu‐Kui

Wang Tianzhen

Wang Wengong

Wang Xiaosheng

Wang Xiao‐zhong

Wang Xiongjun

Wang Yaohe

Wang Yawei

Wang Zhen

Wang Zhirui

Wang Ying

Warfel Noel

Waschke Jens

Watanabe Massayuki

Waterhouse Dale

Watson Zachary

Weigel Ronald

Weiler Hartmut

Wen Jianbo

Werner Martin

Wert Lamas Leon

Westphalen Benedikt

Weyergang Anette

Whitehurst Angelique

Wiemann Stefan

Wikman Harriet

Wikström Pernilla

Wilkinson Simon

Williams Owen

Windle Brad

Wong Alan S.L.

Wu Zijuan

Xie Ruiling

Xing Deyin

Xiong Shunbin

Xu Eugenia Y.

Xu Dawei

Xu Jie

Xu Pinglong

Xu Wenrong

Xu Xin

Xu Zekuan

Yang Henry

Yang Huanjie

Yang Runkuan

Yang Yungui

Yao Bowen

Yao Wantong

Yarden Yosef

Yeh Shuyuan

Yin Ji‐Ye

Ying Jianming

Yochum Gregory

Yoon Jack

Yoshihara Kosuke

Yu Xianjun

Yuan Weitang

Zaman Guido J.R.

Zarrinpar Ali

Zemskova Marina

Zerdes Ioannis

Zhan Qimin

zhang chunlong

Zhang Diancai

Zhang Fan

Zhang Rongxin

Zhang Wei

Zhang Xiaoyang

Zhang Peipei

Zhao Yang

Zhao Yongbing

Zheng Hanqiu

Zhong Jun

Zhou Dong

Zhou Hu

Zhou Meng

Zhu Helene Hu

Zhu J.

Zhu Wencheng

Zhu Xiao

Zhu Xun

Zhuang Likun

Zhuo Wei

Zoni Eugenio

